# Relationships of Residential Distance to Major Traffic Roads with Dementia Incidence and Brain Structure Measures: Mediation Role of Air Pollution

**DOI:** 10.34133/hds.0091

**Published:** 2023-10-16

**Authors:** Chenglong Li, Darui Gao, Yutong Samuel Cai, Jie Liang, Yongqian Wang, Yang Pan, Wenya Zhang, Fanfan Zheng, Wuxiang Xie

**Affiliations:** ^1^ Peking University Clinical Research Institute, Peking University First Hospital, Beijing, China.; ^2^ Key Laboratory of Epidemiology of Major Diseases (Peking University), Ministry of Education, Beijing, China.; ^3^ Centre for Environmental Health and Sustainability, University of Leicester, Leicester, UK.; ^4^ School of Nursing, Peking Union Medical College, Chinese Academy of Medical Sciences, Beijing, China.

## Abstract

**Background:** Uncertainty exists regarding the operating pathways between near-roadway exposure and dementia incidence. We intend to examine relationships between proximity to major roadways with dementia incidence and brain MRI structure measures, and potential mediation roles of air and noise pollution. **Methods:** The cohort study was based on the UK Biobank. Baseline survey was conducted from 2006 to 2010, with linkage to electronic health records conducted for follow-up. Residential distance to major roadways was ascertained residential address postcode. A land use regression model was applied for estimating traffic-related air pollution at residence. Dementia incidence was ascertained using national administrative databases. Brain MRI measures were derived as image-derived phenotypes, including total brain, white matter, gray matter, and peripheral cortical gray matter. ** Results:** We included 460,901 participants [mean (SD) age: 57.1 (8.1) years; men: 45.7%]. Compared with individuals living >1,000 m from major traffic roads, living ≤1,000 m was associated with a 13% to 14% higher dementia risk, accounting for 10% of dementia cases. Observed association between residential distance and dementia was substantially mediated by traffic-related air pollution, mainly nitrogen dioxide (proportion mediated: 63.6%; 95% CI, 27.0 to 89.2%) and PM_2.5_ (60.9%, 26.8 to 87.0%). The shorter residential distance was associated with smaller volumes of brain structures, which was also mediated by traffic-related air pollutants. No significant mediation role was observed of noise pollution. ** Conclusions:** The shorter residential distance to major roads was associated with elevated dementia incidence and smaller brain structure volumes, which was mainly mediated by traffic-related air pollution.

## Introduction

With the rapid increase in the number of aging population, dementia has developed into a serious public health concern, causing a non-neglectable burden on individuals, families, and society [1]. According to Alzheimer’s Disease International, the number of demented people is projected to be 78 million by 2030, with related annual costs estimated at US$2.8 trillion [2]. Without applicable treatment options, dementia prevention remains the top priority, highlighting the need for the identification of potentially modifiable risk factors.

Currently, traffic-related environmental pollutants, such as air pollution, have become a major concern due to their potential roles in the development and pathogenesis of dementia [3]. Epidemiological studies also reported the link between traffic-related air pollution and neurocognitive outcomes, including deteriorated cognitive function, mild cognitive impatient, and dementia incidence [4–7]. In addition, the traffic-related noise exposure has been associated with elevated dementia risk, raising great concern for the public [8,9].

Living near major roadways is indicative of elevated levels of traffic-related pollution and can lead to increased vulnerability to related devastating neurocognitive consequences, which has been indicated by several previous studies [10–12]. However, no study has so far investigated the potential mediating pathways linking traffic proximity exposure with dementia. As living near heavy traffic is regarded as a proxy of traffic-related pollutants and has become a widespread exposure, comprehensively investigating the mediating pathways would provide important indications for target-oriented interventions. It also has been well-established that brain magnetic resonance imaging (MRI) structure measures are closely associated with the incidence and progression of Alzheimer’s disease and could evolve in a complex way throughout aging and the progression of neuropathology, while these measures were rarely evaluated by previous studies in relation to traffic-related exposures [13].

Hence, we aimed at investigating the associations of residential distance to major traffic roads with dementia incidence and brain MRI structure measures. Then, we sought to examine the potential mediating pathways of other traffic-related environmental pollutants, including air and noise pollution. We hypothesized that residential proximity to heavy traffic is associated with elevated dementia risk and reduced brain structure measures, with observed associations partially mediated via traffic-related air and noise pollutants.

## Methods

### Study population

The cohort study was based on the UK Biobank, which enrolled more than 500,000 men and women aged 40 to 69 years from 22 assessment centers in England, Scotland, and Wales during 2006 to 2010. Participants registered at the National Health Service (NHS) were contacted if they lived within 25 miles of an assessment center and recruited after obtaining informed consent. Further details are accessible elsewhere [14,15]. Among the original 502,411 participants available for inclusion, we excluded those who either developed dementia at baseline or had incomplete traffic exposure assessments, yielding an analytical sample of 460,901 participants, with the detailed procedure presented in Fig. S1. Then, we further selected participants with complete follow-up brain MRI structure assessments from the total analytical sample, generating the brain MRI substudy sample of 4,352 individuals.

### Residential distance to major traffic roads

Residential distance to major traffic roads was calculated as the distance (m) between an individual residential address and the nearest major road [16]. The individual residential address was ascertained using a postcode abbreviated of address [17]. After acquiring residential postcodes, the British National Grid coordinates were assigned, based on records from the Ordnance Survey Code Point database covering the UK’s 1.6 million postcodes, which were further rounded to the nearest kilometer to ensure privacy [17]. The major road within the local road network was a road with traffic intensity >5,000 motor vehicles per 24 hours [16]. The traffic intensity was derived from the Road Traffic Statistics Branch at the Department for Transport attached to the local road network [16]. The local road network was taken from the Ordnance Survey Meridian 2 road network (scale 1:50,000, 1-m accuracy) in the year 2009 [16]. Based on a land use regression (LUR) model developed as part of the European Study of Cohorts for Air Pollution Effects (ESCAPE), the residential distance was ascertained using a geographic information system [18]. In addition to the continuous distance measure, we also created five distance categories: less than or equal to 100 m from major traffic roads, 101 to 200 m, 201 to 500 m, 501 to 1,000 m, and more than 1,000 m [19].

### Traffic-related air pollution

We considered three traffic-related air pollution exposures, including nitrogen dioxide, nitrogen oxides, and particulate matter 2.5 (PM_2.5_). Although PM_10_ was not considered a marker of traffic-related air pollution, we also included the pollutant for evaluation, considering its established association with dementia incidence [20]. Using the LUR model, air pollution exposures were estimated for each residential address [21,22]. The LUR model was based on ESCAPE monitoring between 2010 January 26 and 2011 January 18 [21,22]. Therefore, the air pollution estimates represented the annual average exposures for the year 2010. The developed LUR model has been validated with good accuracy for estimating annual average air pollution exposures, with a median explained variance (*R*^2^) of 82% for nitrogen dioxide, 78% for nitrogen oxides, 71% for PM_2.5_, and 77% for PM_10_ [21,22].

### Dementia incidence

Incident dementia cases in UK Biobank were primarily ascertained via two data sources: (a) linked hospital inpatient records from the Hospital Episode Statistics for England, Scottish Morbidity Records for Scotland, and Patient Episode Database for Wales; (b) death register data from NHS Digital [23]. Based on algorithms for outcome ascertainment, individuals with all-cause and cause-specific dementia, including Alzheimer’s disease and vascular dementia, were identified using the International Classification of Diseases Version 9 (ICD-9) and ICD-10 codes (Table S1). According to a validation study, the algorithms developed for ascertaining dementia cases, with data sources of hospital records and death registers, reached positive predictive values of 84.5% for all-cause dementia, 70.8% for Alzheimer’s disease, and 33.3% for vascular dementia [24].

### Brain MRI measure

Brain MRI imaging was conducted as an extension of the UK Biobank study, including the baseline measurement since the year 2014 and the first repeated imaging visit since the year 2019. To account for potential lagged associations of environmental exposures with brain structure measures, we used data from the first repeated imaging visit in 2019 for analysis. Further details regarding image acquisition and processing are available in the protocol [25]. Volumes of specific brain structures, including the total brain, the white matter, the gray matter, and the peripheral cortical gray matter, were made available by the UK Biobank Imaging team as image-derived phenotypes. All volume measures were adjusted for head size using a SIENAX-style analysis [26].

### Covariates

Based on previous studies, we selected established or potential risk factors for dementia incidence or neurocognitive degeneration for adjustment [27,28]. For analyzing dementia incidence, we controlled for socio-demographics, behavioral factors, and clinical characteristics. Socio-demographics included age (years), sex, ethnic background, and education. Behavioral factors included current smoking, alcohol intake, and physical activity. Clinical characteristics included obesity, depressed mood, and the presence of comorbidities including hypertension, diabetes, stroke, and coronary heart disease. For analyzing brain MRI structure measures, we additionally controlled for differences in head positioning inside the scanner (scanner *X*, *Y*, *Z* coordinates), along with the above covariates [29]. Further details regarding covariate definition, assessment, and UK Biobank Data-Field ID (used for searching related variables in the UK Biobank data showcase website: https://biobank.ctsu.ox.ac.uk/showcase/search.cgi) were provided in Table S2.

### Statistical analysis

The mean (SD) or the median [interquartile range (IQR)] was used for summarizing numerical data, and numbers (percentages) were used for summarizing categorical data. Traffic exposures, including both residential distance and air pollution exposures, were standardized using the baseline mean and SD to evaluate associations on a per SD increment scale.

Cox proportional hazard regression was applied to estimate the associations between traffic exposures with dementia incidence, with age as the underlying time scale. For dementia incidence, follow-up years were calculated from the date attending baseline assessment until the date of the first incident dementia diagnosis or death or loss to follow-up, or 2021 December 31, whichever came first. The weighted Schoenfeld residual was used to examine the underlying proportional hazard assumption, and no significant violations for the variables included were observed (*P* > 0.05). Adjusted hazard ratios (HRs) and 95% confidence intervals (CIs) were estimated by building single pollutant models. To quantify the burden of dementia incidence attributed to different traffic exposures, population attributable fraction (PAF) was estimated based on the model-based approach for a time-to-event outcome [30]. Such an approach could derive adjusted PAF estimates from fitted Cox proportional hazards models, with details shown in Supplementary Methods. We defined binary exposures to estimate the corresponding PAF, including residential proximity (≤1,000 m) and nitrogen oxides pollution (≥44.0 μg/m^3^) based on sample distribution, and nitrogen dioxide pollution (≥20.0 μg/m^3^), PM_10_ pollution (≥15.0 μg/m^3^), and PM_2.5_ pollution (≥10.0 μg/m^3^) based on interim targets established by World Health Organization (WHO) air quality guidelines [31]. Linear regression was used to evaluate the associations between traffic exposures with brain MRI structure measures.

The difference method was applied to examine the mediation pathways, with air and noise pollutants included as hypothesized mediators (intermediate variables). Such method estimates the mediation proportion by comparing effect estimates of the exposure variable from models unadjusted and adjusted for the intermediate variable, respectively [32]. The approach has been widely used in literature, can accommodate various type of mediators (binary, ordinal, continuous), and supports Cox regression for analyzing time-to-event outcome [33–35]. The indirect effect is calculated as the difference between the total effect of exposure on the outcome and the effect of exposure adjusted for an intermediate variable (e.g., change in the effect) [32]. The proportion being mediated is calculated as the ratio of the indirect effect with the total effect. We assumed that the rare outcome assumption is met, which was required for making mediation inferences regarding Cox models [32]. Given the relatively low dementia incidence, we confirmed that the assumption was reliable. We also assumed no exposure-mediator interaction [32], which was confirmed by stratified analysis to test for potential interactions between residential distance and air pollutants. After the analysis, we confirmed that the assumption was reliable for air pollutants, except the nitrogen oxides (*P* for interaction: 0.022).

We further conducted several sensitivity analyses. First, we examined the associations between traffic exposures and Alzheimer’s disease and vascular dementia. Second, we excluded dementia cases within 5 years from the air pollution assessment. This part analysis was done to examine the potential lag effect of air pollution on dementia incidence [36]. Third, we excluded participants with stroke. Fourth, to account for the potential regional differences, we additionally fitted a frailty term at the assessment center level for the fitted Cox models. A gamma distribution was assumed for the frailty term. Fifth, we restricted our analysis to participants living at the current address for at least 5 and 10 years, respectively. Sixth, we restricted our analysis to urban residents. Seventh, to examine the mediation role of noise pollution, we additionally controlled for the sound level of noise pollution during the daytime, evening, and nighttime, respectively. Based on a simplified version of the common noise assessment methods in the European Union (CNOSSOS-EU) noise modeling framework, the address-level annual mean road traffic noise level was estimated [37]. The application of CNOSSOS-EU in epidemiological studies has been illustrated, with adequate performance for exposure ranking (Spearman's rank = 0.75) [38]. Eighth, to account for the potential impact of socioeconomic status (SES), we additionally adjusted for two SES variables, including the household income and the multiple deprivation index at the small area level. Ninth, the main analysis was conducted using an inverse probability weighting approach, to further address the potential selection bias regarding brain MRI substudy [39]. The logistic regression was applied to calculate the probability of inclusion, with the same covariates in MRI analysis (except for MRI measuring positions) and traffic exposures included as explanatory variables. Tenth, to examine whether traffic exposures were associated with dementia incidence independently from genetic risk determinants and investigate the potential gene–environment interaction, we additionally controlled for the *APOE* ε4 carrier status and polygenic risk score (PRS) of Alzheimer’s disease [40]. Eleventh, to address the shock of coronavirus disease 2019 (COVID-19) pandemic, we restricted our follow-up to the pre-pandemic period by 2019 December 31. Finally, in addition to the difference method, we further applied the regression-based causal mediation analytical approach [41].

Statistical analysis was conducted using SAS 9.4 (SAS Institute, Cary, NC) and R language 3.6.2 (R Foundation, Vienna, Austria), with a two-tailed alpha of 0.05 considered statistically significant.

## Results

At baseline, the mean (SD) age was 57.1 (8.1) years for the total sample and 53.4 (7.4) years for the brain MRI sample, respectively (Table 1). The median (IQR) follow-up duration was 12.8 (12.1 to 13.4) years, corresponding to 5,736,512 person-years. Among participants of the total sample, 45.7% were men, 93.7% were of White ethnicity, 55.0% had hypertension, and 6.2% had diabetes. The median (IQR) residential distance to major roads was 378.8 (166.7 to 757.6) m among total sample participants, with a mean (SD) annual concentration of 26.7 (7.6) μg/m^3^ for nitrogen dioxide, 44.0 (15.7) μg/m^3^ for nitrogen oxides, 16.2 (1.9) μg/m^3^ for PM_10_, and 10.0 (1.1) μg/m^3^ for PM_2.5_. Among participants of the brain MRI sample, similar baseline characteristics to the total sample were observed (Table 1).

**Table 1.** Baseline characteristics of study participants.

Characteristics	Total study sample (*n* = 460,901)	Brain MRI substudy sample (*n* = 4,352)
Men	210,506 (45.7)	2,074 (47.7)
Age, mean (SD), y	57.1 (8.1)	66.19 (10.11)
Follow-up duration, median (IQR), y	12.8 (12.1–13.4)	12.9 (12.3–13.6)
White	431,945 (93.7)	4,218 (96.9)
Higher education	212,336 (46.1)	2,646 (60.8)
Current smoking	47,755 (10.4)	239 (5.5)
Alcohol intake at least once per week	317,839 (69.0)	3,357 (77.1)
Depressed mood	22,429 (4.9)	121 (2.8)
Physical activity	358,848 (77.9)	3,523 (81.0)
Obesity	112,326 (24.4)	709 (16.3)
Hypertension	253,589 (55.0)	1,807 (41.5)
Diabetes	28,392 (6.2)	101 (2.3)
Stroke	7,697 (1.7)	19 (0.4)
Coronary heart disease	21,065 (4.6)	88 (2.0)
*APOE* ε4 carrier	107,918 (23.4)	983 (22.6)
High dementia polygenic risk	89,180 (19.4)	800 (18.4)
Residential distance to major roads, median (IQR), m	378.8 (166.7–757.6)	378.1 (165.5–746.3)
Nitrogen dioxide, mean (SD), μg/m^3^	26.7 (7.6)	25.6 (7.0)
Nitrogen oxides, mean (SD), μg/m^3^	44.0 (15.7)	42.8 (14.3)
PM_10_, mean (SD), μg/m^3^	16.2 (1.9)	15.9 (1.8)
PM_2.5_, mean (SD), μg/m^3^	10.0 (1.1)	9.9 (1.0)
Peripheral cortical gray matter volume, mean (SD), mm^3^	NA	615,624.1 (40,067.2)
Gray matter volume, mean (SD), mm^3^	NA	791,905.1 (46,654.2)
White matter volume, mean (SD), mm^3^	NA	697,085.8 (40,597.9)
Total brain volume, mean (SD), mm^3^	NA	1,488,990.8 (72,902.8)

IQR, interquartile range; NA, not applicable.

During the follow-up period, a total of 7,000 incident dementia cases were recorded, translating into a crude incidence rate of 10.22/10,000 (95% CI, 10.19 to 10.25) person-years. As shown in Table 2, in comparison with participants living >1,000 m from major roads, individuals living ≤1,000 m had an elevated dementia risk of 13% to 14%. Besides, all four air pollutants were associated with a higher dementia incidence, with each SD increment corresponding to a fully adjusted HR of 1.09 (1.06 to 1.11) for nitrogen dioxide, 1.07 (1.04 to 1.09) for nitrogen oxides, 1.04 (1.01 to 1.06) for PM_10_, and 1.08 (1.06 to 1.11) for PM_2.5_, respectively (Table 2).

**Table 2.** Associations between residential distance to the nearest major road, traffic-related air pollution with dementia incidence.

Traffic exposures	Events/total	Person-years	Crude incidence rate, per 10,000 person-years (95% CI)	HR (95% CI) ^a^	*P*
Residential distance categories					
≤100 m	1,033/66,773	830,410.5	10.24 (10.17–10.32)	1.13 (1.03–1.23)	0.007
101–200 m	1,053/70,482	877,047.9	10.20 (10.13–10.28)	1.13 (1.04–1.23)	0.006
201–500 m	2,145/139,867	1,742,633	10.23 (10.18–10.28)	1.13 (1.05–1.21)	0.002
501–1,000 m	1,711/108,280	1,347,546	10.27 (10.21–10.33)	1.14 (1.05–1.23)	0.001
>1,000 m	1,058/75,499	938,874.6	10.13 (10.06–10.20)	1 (reference)	NA
Residential distance, per SD	7,000/460,901	5,736,512	10.22 (10.19–10.25)	0.94 (0.92–0.97)	0.001
Nitrogen dioxide air pollution, per SD	7,000/460,901	5,736,512	10.22 (10.19–10.25)	1.09 (1.06–1.11)	0.001
Nitrogen oxide air pollution, per SD	7,000/460,901	5,736,512	10.22 (10.19–10.25)	1.07 (1.04–1.09)	0.001
PM_10_ air pollution, per SD	7,000/460,901	5,736,512	10.22 (10.19–10.25)	1.04 (1.01–1.06)	0.003
PM_2.5_ air pollution, per SD	7,000/460,901	5,736,512	10.22 (10.19–10.25)	1.08 (1.06–1.11)	0.001

HR, hazard ratio; CI, confidence interval.

^a^ Cox proportional hazard regression was used to model relationships between traffic exposures and dementia incidence. Age was used as the time scale variable. Adjusted covariates included sex, ethnic background, education, current smoking, alcohol intake, physical activity, obesity, depressed mood, hypertension, diabetes, stroke, and coronary heart disease.

**Fig. 1.**
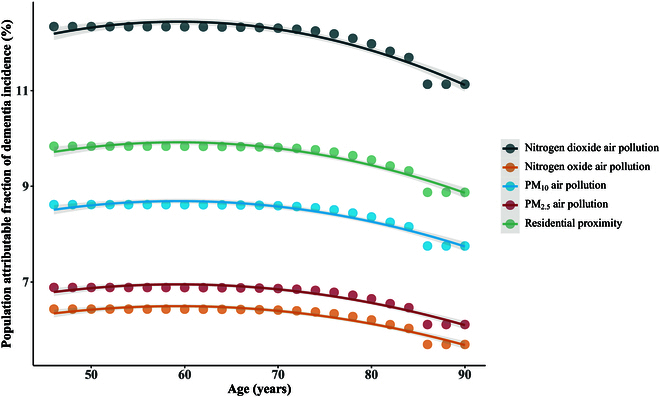
Population attributable fraction of all-cause dementia incidence of residential proximity and traffic-related air pollution exposures. We defined residential proximity (≤1,000 m) and nitrogen oxide pollution (≥44.0 μg/m^3^) based on sample distribution, and nitrogen dioxide pollution (≥20.0 μg/m^3^), PM_10_ pollution (≥15.0 μg/m^3^), and PM_2.5_ pollution (≥10.0 μg/m^3^) based on interim targets established by WHO air quality guidelines. Fitted Cox regression models with age as the time scale were used to estimate the attributable fraction for each exposure, further controlling for sex, ethnic background, education, current smoking, alcohol intake, physical activity, obesity, depressed mood, hypertension, diabetes, stroke, and coronary heart disease. Dots represent point estimates, while lines and shadows represent fitted smooth splines.

As shown in Fig. 1, residential proximity accounted for 8.9% to 9.8% of incident dementia cases. It was also observed that nitrogen dioxide (PAF, 11.1% to 12.3%) was the leading attributable exposure, followed by PM_10_ (PAF, 7.8% to 8.6%), PM_2.5_ (PAF, 6.1% to 6.9%), and nitrogen oxides (PAF, 5.7% to 6.4%).

Among the four brain structure measures, the shorter residential distance to major roads was consistently associated with smaller volumes of the peripheral cortical gray matter, gray matter, and total brain (Table 3). All four air pollutants were associated with smaller volumes of the above brain structures (Table 3).

**Table 3.** Associations between residential distance to the nearest major road, traffic-related air pollution with brain structure measures.

Traffic exposures	Peripheral cortical gray matter volume (mm^3^)		Gray matter volume (mm^3^)		White matter volume (mm^3^)		Total brain volume (mm^3^)	
	β (95% CI) ^a^	*P*	β (95% CI) ^a^	*P*	β (95% CI) ^a^	*P*	β (95% CI) ^a^	*P*
Residential distance categories								
≤100 m (*n* = 627)	−3,871 (−7,224 to −518.8)	0.02	−4,201 (−7,985 to −416.5)	0.03	−4,020 (−8,108 to 67.5)	0.05	−8,221 (−14,573 to −1,869)	0.011
101–200 m (*n* = 679)	−4,824 (−8,102 to −1,546)	0.004	−5,207 (−8,907 to −1,507)	0.006	−5,257 (−9,254 to −1,260)	0.01	−10,464 (−16,675 to −4,253)	0.001
201–500 m (*n* = 1,312)	−1,291 (−4,148 to 1,566.4)	0.38	−1,376 (−4,601 to 1,848.9)	0.40	−2,693 (−6,176 to 791.3)	0.13	−4,069 (−9,482 to 1,344.8)	0.14
501–1,000 m (*n* = 1,042)	−1,435 (−4,414 to 1,545.1)	0.35	−1,675 (−5,039 to 1,688.0)	0.33	−3,435 (−7,069 to 197.8)	0.06	−5,111 (−10,757 to 534.6)	0.08
>1,000 m (*n* = 692)	0 (reference)	NA	0 (reference)	NA	0 (reference)	NA	0 (reference)	NA
Residential distance, per SD	1,653.6 (734.0 to 2,573.1)	0.001	1,736.6 (698.5 to 2,774.6)	0.001	1,076.9 (−44.9 to 2,198.7)	0.06	2,813.4 (107.4 to 4,556.4)	0.002
Nitrogen dioxide air pollution, per SD	−2,156 (−3,163 to −1,149)	0.001	−2,401 (−3,538 to −1,264)	0.001	−1,696 (−2,925 to −467.2)	0.007	−4,097 (−6,005 to −2,189)	0.001
Nitrogen oxide air pollution, per SD	−1,953 (−2,963 to −942.9)	0.001	−2,023 (−3,163 to −882.8)	0.001	−1,225 (−2,457 to 7.8)	0.05	−3,248 (−5,163 to −1,333)	0.001
PM_10_ air pollution, per SD	−1,645 (−2,596 to −693.6)	0.001	−1,918 (−2,991 to −844.8)	0.001	−751.7 (−1,912 to 408.7)	0.20	−2,670 (−4,473 to −867.0)	0.004
PM_2.5_ air pollution, per SD	−1,956 (−2,893 to −1,020)	0.001	−2,006 (−3,063 to −948.8)	0.001	−1,291 (−2,434 to −148.3)	0.03	−3,297 (−5,072 to −1,522)	0.001

^a^ Multivariate linear regression was used to model relationships between traffic exposures and brain MRI measures. Adjusted covariates included age, sex, ethnic background, education, current smoking, alcohol intake, physical activity, obesity, depressed mood, hypertension, diabetes, stroke, coronary heart disease, as well as brain MRI measuring positions.

**Fig. 2.**
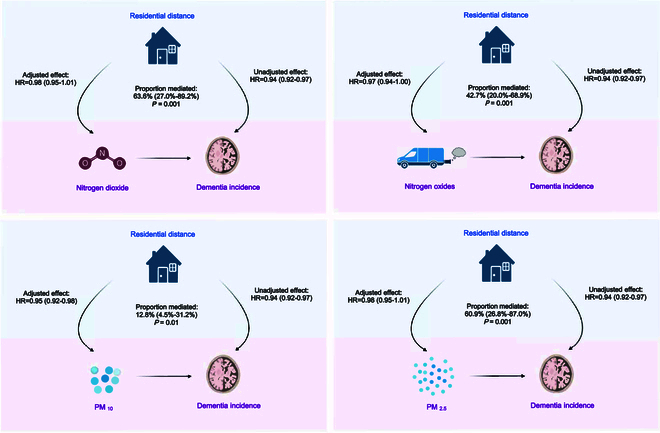
Mediation analysis of the associations between residential distance to major roads with all-cause dementia incidence, by comparing the effect before and after adjusting for the hypothesized mediator of traffic-related air pollution. The unadjusted effect represents the estimate from the Cox regression without adjusting for the hypothesized mediator, while the adjusted effect comes from the Cox regression adjusting for the hypothesized mediator. Four mediation models were built for four air pollutants. Based on the difference between the unadjusted and adjusted effect, the proportion being mediated and hypothesis testing were calculated and conducted for each hypothesized mediator, respectively. Other adjusted covariates were identical to Cox models in Table 2.

All four air pollutants significantly mediated the association between residential distance to major roads with all-cause dementia incidence, as shown in Fig. 2. The nitrogen dioxide mediated 63.6% (95% CI, 27.0 to 89.2%) of the observed association, followed by PM_2.5_ with a mediated proportion of 60.9% (26.8 to 87.0%), nitrogen oxides of 42.7% (20.0 to 68.9%), and PM_10_ of 12.8% (4.5 to 31.2%). Likewise, as shown in Figs. S2 to S4, the four air pollutants all significantly mediated the associations between residential distance to major roads with brain structure measures. Nitrogen dioxide and PM_2.5_ remained the two major mediators of associations between residential distance to major roads and volumes of the peripheral cortical gray matter, gray matter, and total brain, followed by nitrogen oxides and PM_10_.

In sensitivity analyses, the magnitude of associations was not materially altered for either Alzheimer’s disease or vascular dementia (Table S3), when excluding the first 5 years of dementia cases (Table S4), excluding participants with stroke (Table S5), accounting for the spatial correlation within the assessment center (Table S6), restricting to long-term (Tables S7 and S8) and urban residents (Table S9), further controlling for noise pollution (Tables S10 and S11). As shown in Tables S12 and S13, the observed associations between traffic proximity and air pollution with dementia incidence were moderately attenuated after controlling for SES variables, while the associations with brain MRI measures remained evident. The observed associations remained robust after adjusting for selection bias (Table S14) and accounting for *APOE* ε4 carrier status and PRS of Alzheimer’s disease (Tables S15 and S16). No significant gene–environment interactions were observed, except for a stronger association between PM_10_ and dementia incidence observed among the non-*APOE* ε4 carrier than the *APOE* ε4 carrier (*P* = 0.03 for interaction), as shown in Tables S17 and S18. After restricting to the pre-pandemic follow-up, our main findings remained evident (Table S19). As shown in Table S20, the regression-based causal mediation analysis showed similar findings, with nitrogen dioxide and PM_2.5_ identified as two major mediators.

## Discussion

We found that traffic proximity exposure was consistently associated with the higher dementia incidence and smaller brain structure volumes. The observed associations remained evident after controlling for other related factors and robust to various sensitivity analyses. Despite the observed associations being moderate, PAF analysis showed that traffic proximity exposure accounted for up to 9.8% of dementia incidence burden. Further mediation analysis identified traffic-related air pollution as the main operating mechanism linking residential distance with dementia and brain structure measures, with nitrogen dioxide and PM_2.5_ consistently identified as two major mediators. By contrast, no significant mediation role was observed for noise pollution. To our knowledge, this is the first prospective study simultaneously investigating the associations of traffic proximity with dementia incidence and brain structure measures, as well as the mediation pathways behind the associations.

Previous studies have linked residential proximity to traffic with neurocognitive outcomes, while significant gaps remained in the understanding of the potential operating mechanism. In the Framingham Offspring Study of 943 community-dwelling older people with brain MRI structure measures, the further distance from a major roadway was associated with a greater log-transformed white matter hyperintensity volume [10]. A population-based cohort study also examined the association between residential proximity to major roadways and dementia incidence [12]. The study observed a modest attenuation when traffic-related air pollutants including nitrogen dioxide and PM_2.5_ were additionally adjusted in the models [12]. Based on the study, we not only found that traffic proximity was associated with elevated dementia incidence but also confirmed that the observed association was mainly mediated via the pathway of traffic-related air pollution, especially of the pollutants of nitrogen dioxide and PM_2.5_.

Notably, previous studies have associated long-term traffic noise pollution with the higher dementia incidence [9], while no significant mediation effect was observed for noise pollution in our study. In addition, traffic proximity also represents heightened exposure to traffic-related pollutants other than air and noise pollution, such as heavy metals, polycyclic aromatic hydrocarbons, and volatile organic compounds [12]. Interestingly, we found that the associations between traffic proximity and dementia incidence also became attenuated when SES variables were additionally adjusted. Further investigations are warranted to validate our findings and examine the potential roles of other traffic-related environmental pollutants, as well as SES inequity.

We also found that traffic proximity was consistently associated with smaller volumes of brain structures including peripheral cortical gray matter and gray matter, all of which were associated with pathological changes in Alzheimer’s disease [42,43]. Considering that brain structure measures at the pre-symptomatic stage provide important indications regarding dementia onset and progression [25], our findings showed that traffic exposures could serve as early risk factors for dementia, highlighting the need for preventive measures.

Although our study indicates that traffic-related air pollution could be the main operating mechanism linking traffic proximity with dementia incidence and brain structure changes, further investigations are still warranted to examine the underlying biological mechanisms. First, according to previous studies, systemic inflammation could be an important pathway. Heightened air pollution exposure, arising from traffic proximity, represents elevated exposed levels to particulates and diesel exhaust, which could further provoke the oxidative stress and systemic inflammatory responses, as well as activate the microglia in the brain [44]. Second, ultrafine particle fraction has also been found to be associated with detrimental health consequences of exposure to PM_10_ or PM_2.5_ [45]. Modern fossil fuel combustion technologies were also responsible for excessive emission of ultrafine particles, which can in turn contribute to the absorption of reactive oxygen species, enhancing the toxicity of ultrafine particles [46]. Previous studies also found that ultrafine particles could penetrate the blood–brain barrier and enter into the olfactory bulb and the frontal cortical areas of brain [44].

Our study provides two implications. First, it indicates the potential significance of reducing traffic proximity exposure for preventing dementia onset and progression at the early pre-symptomatic stage. In the context of the widespread traffic proximity exposure and ever-expanding dementia-related disease burden, the indication is crucial for developing environmental health policies and land use planning. Second, for the first time, it highlights traffic-related air pollution as the main mediating pathway linking traffic proximity and neurocognitive outcomes, instead of noise pollution. This indication is also important. Currently, the number of people living near traffic is surging, posing serious challenges for developing policies. Our findings reveal that, in addition to eliminating traffic proximity exposure, tackling traffic-related air pollution may serve as an alternative option. Considering that living near major traffic roads also represents heightened exposure to relevant pollutants, more target-oriented preventive measures would be relevant. According to our analysis, future actions should focus on air pollutants including nitrogen dioxide and PM_2.5_. On the one hand, the two pollutants were major attributable exposures to dementia incidence burden, accounting for up to 12.3% of dementia cases. On the other hand, mediation analysis consistently identified the two pollutants as leading mediators, explaining up to 63.6% and 39.9% of the associations between traffic proximity with dementia incidence and brain structure measures, respectively. It should be noted that the associations we observed do not imply causation. Further investigations capable of determining causal relations are warranted to confirm our findings.

There were several strengths of the study. First, with the large sample-size data and long follow-up duration, we were able to examine the prospective associations robustly. Second, based on the national registries, the dementia case ascertainment procedure in our study had better reliability, compared to merely relying on the patient-reported diagnosis. We were also able to identify the incidence of different dementia subtypes including Alzheimer’s disease and vascular dementia, enabling more comprehensive evaluations. Third, we incorporated the brain MRI structure measures into assessments, which could provide indications regarding pathological changes of Alzheimer’s disease at the early pre-symptomatic stage [42,43]. Fourth, we accounted for various traffic exposures, which were evaluated at a fine scale. We evaluated the residential proximity to traffic after considering the traffic intensity of the nearest roadways. The LUR model used for estimating air pollution exposures also included traffic variables as predictors, such as traffic intensity, heavy traffic load, and road length [21]. Thus, the estimated traffic-related air pollution exposures could represent the potential impact of traffic emissions. We also considered the sound level of noise pollution. Finally, our findings were robust. After controlling for several important factors and conducting several sensitivity analyses including further accounting for genetic dementia risk determinants, the observed associations remained evident.

Several limitations should be noted. First, the residential address was ascertained using a postcode. Therefore, we could not obtain the exact exposed level for the individual. That might be the reason why the HRs of dementia incidence were similar across different distance categories. The less accurate residential address might also diminish the reliability of air pollution estimates, such as nitrogen oxides. In addition, we also found the lack of gradient of the noise exposure level between different distance categories, which might be the reason for the insignificant mediation effect of noise pollution. Second, only 1-year air pollutant surveillance data were used for evaluation, which might fail to reflect the long-term air pollution exposure and the associations with dementia incidence. Additionally, the noise exposure level was also evaluated using 1-year monitoring data, which failed to capture the long-term exposure and the associations with dementia incidence. A previous study reporting positive associations between noise exposure and dementia incidence evaluated residential noise exposure based on monitoring data from 1995 to 2015 (8). Thus, the lack of long-term noise monitoring data might be another reason for the insignificant mediation effect of noise pollution. Third, most participants were of white ethnicity, diminishing the generalizability of our findings. Fourth, due to data accessibility, we could not consider regional differences in dementia incidence. Although we conducted a sensitivity analysis accounting for the potential spatial correlations at the assessment center level, it still could not eliminate all other facets of regional heterogeneity. Fifth, it should be noted that the dementia ascertainment data sources did not include the primary care records. Therefore, the dementia incidence might be underestimated in the current study. In addition, the dementia ascertainment algorithms showed a much lower positive predictive value for identifying vascular dementia cases, which could undermine the reliability of our findings regarding the dementia subtype. Sixth, the underlying no exposure-mediator assumption for the mediation analysis was slightly violated for nitrogen oxides. However, previous studies also showed that violation of the assumption did not bring a substantial impact on the mediation proportion [47]. Hence, our primary findings could remain robust to the violation. Seventh, the LUR model did not account for the role of prevailing winds, undermining the reliability of estimates of traffic proximity and air pollutants. Finally, residual confounding still could not be precluded, preventing us from drawing causal conclusions [48].

## Conclusion

In conclusion, we found that the shorter residential distance to major traffic roads was consistently associated with the higher dementia risk and smaller brain structure volumes. Traffic-related air pollution was identified as the main mediating pathway linking traffic proximity and neurocognitive outcomes, rather than noise pollution. These findings support tackling traffic-related air pollution as the key to preventing dementia onset among people living near heavy traffic.

## Data Availability

The data that support the findings of this study are available from the UK Biobank project site, subject to registration and application process. Further details can be found at https://www.ukbiobank.ac.uk.
