# Supplementary material for: Relationships of Residential Distance to Major Traffic Roads with Dementia Incidence and Brain Structure Measures: Mediation Role of Air Pollution
Source: Health Data Sci. 2023 Oct 16;3:0091. doi: 10.34133/hds.0091 (PMC10880167; doi:10.34133/hds.0091)
Supplement: Supplementary 1 — Supplementary Methods Tables S1 to S20 Figs. S1 to S4 [file hds.0091.f1.docx]

**Supplemental Methods**

**1. Population attributable fraction estimation**

We used the “AFcoxph” function provided in the R package “AF” to estimate the population attributable fraction (PAF) function based on a fitted Cox proportional hazard regression model for each of the traffic exposures. The approach estimates the PAF for a time-to-event outcome under the hypothetical scenario where a binary exposure is eliminated from the population. The detailed calculation was summarized below:

$$PAF=1-\frac{\left\{ 1-S_{0}(t) \right\}}{\left\{ 1-S(t) \right\}}$$

The $S_{0}(t)$ represents the counterfactual survival function for the event if the binary exposure would have been eliminated from the population at baseline, while the $S\left( t \right)$ represents the factual survival function. The $t$ represents the time scale variable, e.g. the age in years. The function uses a fitted Cox proportional hazards regression model to estimate the $S\left( t \right)$ after adjusting for the covariates included in the model.

**Online only tables and figures**

[**Appendix Table 1.** ICD-9 & ICD-10 codes used to ascertain all-cause and cause-specific dementia cases.](#_Toc140777822)

[**Appendix Table 2.** Covariates definition and assessment.](#_Toc140777823)

[**Appendix Table 3.** Associations between residential distance to the nearest major road, traffic-related air pollution with cause-specific dementia incidence.](#_Toc140777824)

[**Appendix Table 4.** Associations between residential distance to the nearest major road, traffic-related air pollution with dementia incidence, further excluding dementia cases within five years from assessment of residential air pollution.](#_Toc140777825)

[**Appendix Table 5.** Associations between residential distance to the nearest major road, traffic-related air pollution with dementia incidence, further excluding participants with previous stroke at baseline assessment.](#_Toc140777826)

[**Appendix Table 6.** Associations between residential distance to the nearest major road, traffic-related air pollution with dementia incidence, further accounting for clustering structure at the assessment center level.](#_Toc140777827)

[**Appendix Table 7.** Associations between residential distance to the nearest major road, traffic-related air pollution with dementia incidence, restricted to participants living at the current address for at least five years.](#_Toc140777828)

[**Appendix Table 8.** Associations between residential distance to the nearest major road, traffic-related air pollution with dementia incidence, restricted to participants living at the current address for at least ten years.](#_Toc140777829)

[**Appendix Table 9.** Associations between residential distance to the nearest major road, traffic-related air pollution with dementia incidence, restricted to urban residents.](#_Toc140777830)

[**Appendix Table 10.** Association between residential distance to the nearest major road and dementia incidence, further controlling for noise pollution.](#_Toc140777831)

[**Appendix Table 11.** Associations between residential distance to the nearest major road and brain structure measures, further controlling for noise pollution.](#_Toc140777832)

[**Appendix Table 12.** Association between residential distance to the nearest major road, traffic-related air pollution with dementia incidence, further controlling for socioeconomic status variables.](#_Toc140777833)

[**Appendix Table 13.** Associations between residential distance to the nearest major road and brain structure measures, further controlling for socioeconomic status variables.](#_Toc140777834)

[**Appendix Table 14.** Associations between residential distance to the nearest major road and brain structure measures, based on the IPW sample.](#_Toc140777835)

[**Appendix Table 15.** Association between residential distance to the nearest major road, traffic-related air pollution with dementia incidence, further controlling for *APOE* ε4 carrier status.](#_Toc140777836)

[**Appendix Table 16.** Association between residential distance to the nearest major road, traffic-related air pollution with dementia incidence, further controlling for dementia polygenic risk category.](#_Toc140777837)

[**Appendix Table 17.** Association between residential distance to the nearest major road, traffic-related air pollution with dementia incidence, further stratified by *APOE* ε4 carrier status.](#_Toc140777838)

[**Appendix Table 18.** Association between residential distance to the nearest major road, traffic-related air pollution with dementia incidence, further stratified by dementia polygenic risk category.](#_Toc140777839)

[**Appendix Table 19.** Association between residential distance to the nearest major road, traffic-related air pollution with dementia incidence, further restricting to the pre-pandemic follow-up period.](#_Toc140777840)

[**Appendix Table 20.** Causal mediation analysis of the associations between residential distance to major roads with all-cause dementia incidence, estimated using the regression-based approach.](#_Toc140777841)

**Appendix Figure 1.** Participants selection diagram.

**Appendix Figure 2.** Mediation analysis of the associations between residential distance to major roads with volume of peripheral cortical grey matter, by comparing the effect before and after adjusting for the hypothesized mediator of traffic-related air pollution.

**Appendix Figure 3.** Mediation analysis of the associations between residential distance to major roads with volume of grey matter, by comparing the effect before and after adjusting for the hypothesized mediator of traffic-related air pollution.

**Appendix Figure 4.** Mediation analysis of the associations between residential distance to major roads with volume of total brain, by comparing the effect before and after adjusting for the hypothesized mediator of traffic-related air pollution.

**Appendix Table 1. ICD-9 & ICD-10 codes used to ascertain all-cause and cause-specific dementia cases.**

| **ICD-9 codes** | | | | | |
| --- | --- | --- | --- | --- | --- |
| Code type | ICD-9 code | ICD-9 text | Alzheimer’s disease | Vascular dementia | All-cause dementia |
| ICD-9 code | 290.2 | Senile dementia, depressed or paranoid type |  |  | √ |
| ICD-9 code | 290.3 | Senile dementia with acute confusional state |  |  | √ |
| ICD-9 code | 290.4 | Arteriosclerotic dementia |  | √ | √ |
| ICD-9 code | 291.2 | Other alcoholic dementia |  |  | √ |
| ICD-9 code | 294.1 | Dementia in other conditions classified elsewhere |  |  | √ |
| ICD-9 code | 331.0 | Alzheimer's disease | √ |  | √ |
| ICD-9 code | 331.1 | Pick's disease |  |  | √ |
| ICD-9 code | 331.2 | Senile degeneration of brain |  |  | √ |
| ICD-9 code | 331.5 | Creutzfeldt-Jakob disease |  |  | √ |
| **ICD-10 codes** | | | | | |
| Code type | ICD-10 code | ICD-10 text | Alzheimer’s disease | Vascular dementia | All-cause dementia |
| ICD-10 code | A81.0 | Sporadic Creutzfeldt-Jakob disease |  |  | √ |
| ICD-10 code | F00 | Dementia in Alzheimer's disease | √ |  | √ |
| ICD-10 code | F00.0 | Dementia in Alzheimer's disease with early onset | √ |  | √ |
| ICD-10 code | F00.1 | Dementia in Alzheimer's disease with late onset | √ |  | √ |
| ICD-10 code | F00.2 | Dementia in Alzheimer's disease, atypical or mixed type | √ |  | √ |
| ICD-10 code | F00.9 | Dementia in Alzheimer's disease, unspecified | √ |  | √ |
| ICD-10 code | F01 | Vascular dementia |  | √ | √ |
| ICD-10 code | F01.0 | Vascular dementia of acute onset |  | √ | √ |
| ICD-10 code | F01.1 | Multi-infarct dementia |  | √ | √ |
| ICD-10 code | F01.2 | Subcortical vascular dementia |  | √ | √ |
| ICD-10 code | F01.3 | Mixed cortical and sub-cortical vascular dementia |  | √ | √ |
| ICD-10 code | F01.8 | Other vascular dementia |  | √ | √ |
| ICD-10 code | F01.9 | Vascular dementia, unspecified |  | √ | √ |
| ICD-10 code | F02 | Dementia in other diseases classified elsewhere |  |  | √ |
| ICD-10 code | F02.0 | Dementia in Picks disease |  |  | √ |
| ICD-10 code | F02.1 | Dementia in Creutzfeldt-Jacob disease |  |  | √ |
| ICD-10 code | F02.2 | Dementia in Huntington’s disease |  |  | √ |
| ICD-10 code | F02.3 | Dementia in Parkinson’s disease |  |  | √ |
| ICD-10 code | F02.4 | Dementia in HIV disease |  |  | √ |
| ICD-10 code | F02.8 | Dementia in other specified diseases classified elsewhere |  |  | √ |
| ICD-10 code | F03 | Unspecified dementia |  |  | √ |
| ICD-10 code | F05.1 | Delirium superimposed on dementia |  |  | √ |
| ICD-10 code | F10.6 | Mental and behavioural disorders due to use of alcohol - amnesic syndrome |  |  | √ |
| ICD-10 code | G30 | Alzheimer’s disease | √ |  | √ |
| ICD-10 code | G30.0 | Alzheimer’s disease with early onset | √ |  | √ |
| ICD-10 code | G30.1 | Alzheimer’s disease with late onset | √ |  | √ |
| ICD-10 code | G30.8 | Other Alzheimer's disease | √ |  | √ |
| ICD-10 code | G30.9 | Alzheimer's disease unspecified | √ |  | √ |
| ICD-10 code | G31.0 | Circumscribed brain atrophy |  |  | √ |
| ICD-10 code | G31.1 | Senile degeneration of brain |  |  | √ |
| ICD-10 code | G31.8 | Other specified degenerative diseases of  nervous system |  |  | √ |
| ICD-10 code | I67.3 | Binswanger’s disease |  | √ |  |

**Appendix Table 2. Covariates definition and assessment.**

| **Covariates** | **Definition** | **Assessment** | **UK Biobank** **Data-Field ID** |
| --- | --- | --- | --- |
| Age (years) | Age in years | Difference between date attended baseline assessment and date of birth recorded by NHS | 21003 |
| Sex | Men, Women | NHS derived and/or touchscreen questionnaire | 31 |
| Ethnic background | White, Non-White (Mixed, Asian, Black, Chinese, Other) | Touchscreen questionnaire: “What is your ethnic group?” | 21000 |
| Education | Higher education (college or university degree, other professional qualifications), other than higher education | Touchscreen questionnaire: “Which of the following qualifications do you have?” | 6138 |
| Current smoking | Yes, No | Touchscreen questionnaire: “Do you smoke tobacco now?” and “In the past, how often have you smoked tobacco?” | 20116 |
| Alcohol intake | At least once per week, less than once per week | Touchscreen questionnaire: “About how often do you drink alcohol?” | 1558 |
| Obesity | Yes (BMI ≥ 30 kg/m^2^), No (BMI < 30 kg/m^2^) | Physical examination: body mass index | 21001 |
| Physical activity | Attending moderate or vigorous physical activity 10+ minutes at least twice per week, less than twice per week | Touchscreen questionnaire: “In a typical WEEK, on how many days did you do 10 minutes or more of moderate physical activities like carrying light loads, cycling at normal pace? (Do not include walking);  In a typical WEEK, how many days did you do 10 minutes or more of vigorous physical activity? (These are activities that make you sweat or breathe hard such as fast cycling, aerobics, heavy lifting)” | 884, 904 |
| Depressed mood | Yes (nearly every day or more than half the days), No (not at all or several days) | Touchscreen questionnaire: “Over the past two weeks, how often have you felt down, depressed or hopeless?” | 2050 |
| Hypertension | Yes, No | Touchscreen questionnaire and verbal interview: self-reported hypertension or anti-hypertensive medication use;  Average SBP/DBP ≥ 140/90 mmHg at baseline | 6150, 20002, 6177, 4079, 4080, 93, 94 |
| Diabetes | Yes, No | Touchscreen questionnaire and verbal interview: self-reported diabetes (diabetes, type 1 diabetes or type 2 diabetes) or medication use for lowering blood glucose;  Plasma hba1c ≥ 48 mmol/mol (6.5%) | 2443, 20002, 6153, 6177, 30750, 20003 |
| Stroke | Yes, No | Touchscreen questionnaire and verbal interview: self-reported previous stroke or transient ischaemic attack (TIA) | 6150, 20002 |
| Coronary heart disease | Yes, No | Touchscreen questionnaire and verbal interview: self-reported angina, heart attack/myocardial infarction | 6150, 20002 |
| Brain MRI measuring positions | Scanner lateral (X) brain position, Scanner transverse (Y) brain position, Scanner longitudinal (Z) brain position | Brain MRI imaging | 25756, 25757, 25758 |

**Appendix Table 3. Associations between residential distance to the nearest major road, traffic-related air pollution with cause-specific dementia incidence.**

| **Traffic exposures, per SD ^*^** | **Cause-specific dementia incidence** | | |
| --- | --- | --- | --- |
|  | HR | 95% CI | *P* |
| **Alzheimer’s disease (case/n=2927/460 901)** | | | |
| Residential distance to the nearest major road | 0.95 | 0.91-0.99 | 0.04 |
| Nitrogen dioxide air pollution | 1.09 | 1.05-1.13 | 0.001 |
| Nitrogen oxides air pollution | 1.08 | 1.04-1.12 | 0.001 |
| PM_10_ air pollution | 1.04 | 1.01-1.08 | 0.02 |
| PM_2.5_ air pollution | 1.10 | 1.06-1.14 | 0.001 |
| **Vascular dementia (case/n=1545/460 901)** | | | |
| Residential distance to the nearest major road | 0.92 | 0.86-0.99 | 0.02 |
| Nitrogen dioxide air pollution | 1.09 | 1.04-1.15 | 0.001 |
| Nitrogen oxides air pollution | 1.06 | 1.01-1.12 | 0.010 |
| PM_10_ air pollution | 1.08 | 1.03-1.13 | 0.003 |
| PM_2.5_ air pollution | 1.07 | 1.02-1.13 | 0.004 |

**^*^** Traffic exposures were analyzed on the scale of per SD increment. Cox proportional hazard regression was used to model relationships between Traffic exposures and dementia incidence. Age was used as the time scale variable. Adjusted covariates included sex, ethnic background, education, current smoking, alcohol intake, physical activity, obesity, depressed mood, hypertension, diabetes, stroke, and coronary heart disease.

**Appendix Table 4. Associations between residential distance to the nearest major road, traffic-related air pollution with dementia incidence, further excluding dementia cases within five years from assessment of residential air pollution.**

| **Traffic exposures, per SD ^*^** | **Dementia incidence (case/n=5788/448 037)** | | |
| --- | --- | --- | --- |
|  | HR | 95% CI | *P* |
| Residential distance to the nearest major road | 0.95 | 0.92-0.98 | 0.001 |
| Nitrogen dioxide air pollution | 1.08 | 1.06-1.11 | 0.001 |
| Nitrogen oxides air pollution | 1.06 | 1.03-1.09 | 0.001 |
| PM_10_ air pollution | 1.03 | 1.01-1.06 | 0.013 |
| PM_2.5_ air pollution | 1.08 | 1.05-1.10 | 0.001 |

**^*^** Traffic exposures were analyzed on the scale of per SD increment. Cox proportional hazard regression was used to model relationships between Traffic exposures and dementia incidence. Age was used as the time scale variable. Adjusted covariates included sex, ethnic background, education, current smoking, alcohol intake, physical activity, obesity, depressed mood, hypertension, diabetes, stroke, and coronary heart disease.

**Appendix Table 5. Associations between residential distance to the nearest major road, traffic-related air pollution with dementia incidence, further excluding participants with previous stroke at baseline assessment.**

| **Traffic exposures, per SD ^*^** | **Dementia incidence (case/n=6570/453 204)** | | |
| --- | --- | --- | --- |
|  | HR | 95% CI | *P* |
| Residential distance to the nearest major road | 0.94 | 0.92-0.97 | 0.001 |
| Nitrogen dioxide air pollution | 1.09 | 1.06-1.11 | 0.001 |
| Nitrogen oxides air pollution | 1.06 | 1.04-1.09 | 0.001 |
| PM_10_ air pollution | 1.04 | 1.01-1.06 | 0.003 |
| PM_2.5_ air pollution | 1.08 | 1.06-1.11 | 0.001 |

**^*^** Traffic exposures were analyzed on the scale of per SD increment. Cox proportional hazard regression was used to model relationships between Traffic exposures and dementia incidence. Age was used as the time scale variable. Adjusted covariates included sex, ethnic background, education, current smoking, alcohol intake, physical activity, obesity, depressed mood, hypertension, diabetes, stroke, and coronary heart disease.

**Appendix Table 6. Associations between residential distance to the nearest major road, traffic-related air pollution with dementia incidence, further accounting for clustering structure at the assessment center level.**

| **Traffic exposures, per SD ^*^** | **Dementia incidence (case/n=7000/460 901)** | | |
| --- | --- | --- | --- |
|  | HR | 95% CI | *P* |
| Residential distance to the nearest major road | 0.95 | 0.92-0.98 | 0.001 |
| Nitrogen dioxide air pollution | 1.10 | 1.07-1.13 | 0.001 |
| Nitrogen oxides air pollution | 1.06 | 1.04-1.09 | 0.001 |
| PM_10_ air pollution | 1.03 | 1.01-1.06 | 0.008 |
| PM_2.5_ air pollution | 1.09 | 1.06-1.11 | 0.001 |

**^*^** Traffic exposures were analyzed on the scale of per SD increment. Cox proportional hazard regression was used to model relationships between Traffic exposures and dementia incidence. Age was used as the time scale variable. Adjusted covariates included sex, ethnic background, education, current smoking, alcohol intake, physical activity, obesity, depressed mood, hypertension, diabetes, stroke, and coronary heart disease.

**Appendix Table 7. Associations between residential distance to the nearest major road, traffic-related air pollution with dementia incidence, restricted to participants living at the current address for at least five years.**

| **Traffic exposures, per SD ^*^** | **Dementia incidence (case/n=6024/387 226)** | | |
| --- | --- | --- | --- |
|  | HR | 95% CI | *P* |
| Residential distance to the nearest major road | 0.95 | 0.92-0.98 | 0.002 |
| Nitrogen dioxide air pollution | 1.09 | 1.07-1.12 | 0.001 |
| Nitrogen oxides air pollution | 1.07 | 1.04-1.09 | 0.001 |
| PM_10_ air pollution | 1.03 | 1.01-1.06 | 0.008 |
| PM_2.5_ air pollution | 1.09 | 1.06-1.12 | 0.001 |

**^*^** Traffic exposures were analyzed on the scale of per SD increment. Cox proportional hazard regression was used to model relationships between Traffic exposures and dementia incidence. Age was used as the time scale variable. Adjusted covariates included sex, ethnic background, education, current smoking, alcohol intake, physical activity, obesity, depressed mood, hypertension, diabetes, stroke, and coronary heart disease.

**Appendix Table 8. Associations between residential distance to the nearest major road, traffic-related air pollution with dementia incidence, restricted to participants living at the current address for at least ten years.**

| **Traffic exposures, per SD ^*^** | **Dementia incidence (case/n=5151/311 296)** | | |
| --- | --- | --- | --- |
|  | HR | 95% CI | *P* |
| Residential distance to the nearest major road | 0.96 | 0.92-0.99 | 0.010 |
| Nitrogen dioxide air pollution | 1.08 | 1.05-1.11 | 0.001 |
| Nitrogen oxides air pollution | 1.06 | 1.03-1.09 | 0.001 |
| PM_10_ air pollution | 1.03 | 1.00-1.05 | 0.07 |
| PM_2.5_ air pollution | 1.08 | 1.05-1.11 | 0.001 |

**^*^** Traffic exposures were analyzed on the scale of per SD increment. Cox proportional hazard regression was used to model relationships between Traffic exposures and dementia incidence. Age was used as the time scale variable. Adjusted covariates included sex, ethnic background, education, current smoking, alcohol intake, physical activity, obesity, depressed mood, hypertension, diabetes, stroke, and coronary heart disease.

**Appendix Table 9. Associations between residential distance to the nearest major road, traffic-related air pollution with dementia incidence, restricted to urban residents.**

| **Traffic exposures, per SD ^*^** | **Dementia incidence (case/n=6100/392 058)** | | |
| --- | --- | --- | --- |
|  | HR | 95% CI | *P* |
| Residential distance to the nearest major road | 0.94 | 0.91-0.98 | 0.005 |
| Nitrogen dioxide air pollution | 1.07 | 1.04-1.10 | 0.001 |
| Nitrogen oxides air pollution | 1.05 | 1.02-1.08 | 0.001 |
| PM_10_ air pollution | 1.02 | 1.00-1.05 | 0.10 |
| PM_2.5_ air pollution | 1.07 | 1.04-1.10 | 0.001 |

**^*^** Traffic exposures were analyzed on the scale of per SD increment. Cox proportional hazard regression was used to model relationships between Traffic exposures and dementia incidence. Age was used as the time scale variable. Adjusted covariates included sex, ethnic background, education, current smoking, alcohol intake, physical activity, obesity, depressed mood, hypertension, diabetes, stroke, and coronary heart disease.

**Appendix Table 10.** **Association between residential distance to the nearest major road and dementia incidence, further controlling for** **noise pollution.**

| **Traffic exposures, per SD ^*^** | **Dementia incidence (case/n=7000/460 901)** | | |
| --- | --- | --- | --- |
|  | HR | 95% CI | *P* |
| Residential distance to the nearest major road | 0.94 | 0.92-0.97 | 0.001 |
| Further controlling for average daytime noise pollution | 0.93 | 0.91-0.96 | 0.001 |
| Further controlling for average evening noise pollution | 0.93 | 0.91-0.96 | 0.001 |
| Further controlling for average night-time noise pollution | 0.93 | 0.91-0.96 | 0.001 |

**^*^** Traffic exposures were analyzed on the scale of per SD increment. Cox proportional hazard regression was used to model relationships between traffic exposures and dementia incidence. Age was used as the time scale variable. Adjusted covariates included sex, ethnic background, education, current smoking, alcohol intake, physical activity, obesity, depressed mood, hypertension, diabetes, stroke, and coronary heart disease.

**Appendix Table 11. Associations between residential distance to the nearest major road and brain structure measures, further controlling for noise pollution.**

| **Traffic exposures, per SD ^*^** | **Peripheral cortical grey matter volume (mm^3^)** | | | **Grey matter**  **volume (mm^3^)** | | | **White matter**  **volume (mm^3^)** | | | **Total brain**  **volume (mm^3^)** | | |
| --- | --- | --- | --- | --- | --- | --- | --- | --- | --- | --- | --- | --- |
|  | β | 95% CI | *P* | β | 95% CI | *P* | β | 95% CI | *P* | β | 95% CI | *P* |
| Residential distance to the nearest major road | 1653.6 | 734.0 to 2573.1 | 0.001 | 1736.6 | 698.5 to 2774.6 | 0.001 | 1076.9 | -44.9 to 2198.7 | 0.06 | 2813.4 | 1070.4 to 4556.4 | 0.002 |
| Further controlling for average daytime noise pollution | 1645.4 | 709.8 to 2581.0 | 0.001 | 1719.3 | 663.1 to 2775.5 | 0.001 | 1148.1 | 6.7 to 2289.4 | 0.05 | 2867.3 | 1093.8 to 4640.7 | 0.002 |
| Further controlling for average evening noise pollution | 1645.2 | 709.6 to 2580.9 | 0.001 | 1719.0 | 662.8 to 2775.2 | 0.001 | 1148.2 | 6.8 to 2289.5 | 0.05 | 2867.1 | 1093.7 to 4640.6 | 0.002 |
| Further controlling for average night-time noise pollution | 1645.4 | 709.8 to 2581.0 | 0.001 | 1719.3 | 663.1 to 2775.5 | 0.001 | 1148.0 | 6.7 to 2289.4 | 0.05 | 2867.3 | 1093.8 to 4640.7 | 0.002 |

**^*^** Traffic exposures were analyzed on the scale of per SD increment. Multivariate linear regression was used to model relationships between traffic exposures and brain MRI measures. Adjusted covariates included age, sex, ethnic background, education, current smoking, alcohol intake, physical activity, obesity, depressed mood, hypertension, diabetes, stroke, coronary heart disease, as well as brain MRI measuring positions.

**Appendix Table 12. Association between residential distance to the nearest major road, traffic-related air pollution with dementia incidence, further controlling for socioeconomic status variables.**

| **Traffic exposures, per SD ^*^** | **Dementia incidence (case/n=6821/448 442)** | | |
| --- | --- | --- | --- |
|  | HR | 95% CI | *P* |
| Residential distance to the nearest major road | 0.96 | 0.94-0.99 | 0.02 |
| Nitrogen dioxide air pollution | 1.04 | 1.01-1.07 | 0.004 |
| Nitrogen oxides air pollution | 1.02 | 1.00-1.05 | 0.06 |
| PM_10_ air pollution | 1.02 | 1.00-1.04 | 0.11 |
| PM_2.5_ air pollution | 1.03 | 1.01-1.06 | 0.009 |

**^*^** Traffic exposures were analyzed on the scale of per SD increment. Cox proportional hazard regression was used to model relationships between traffic exposures and dementia incidence. Age was used as the time scale variable. Adjusted covariates included sex, ethnic background, education, current smoking, alcohol intake, physical activity, obesity, depressed mood, hypertension, diabetes, stroke, and coronary heart disease. Socioeconomic status variables including the household income and the multiple deprivation index at the at the small area level were also adjusted.

**Appendix Table 13. Associations between residential distance to the nearest major road and brain structure measures, further controlling for socioeconomic status variables.**

| **Traffic exposures, per SD ^*^** | **Peripheral cortical grey matter volume (mm^3^)** | | | **Grey matter**  **volume (mm^3^)** | | | **White matter**  **volume (mm^3^)** | | | **Total brain**  **volume (mm^3^)** | | |
| --- | --- | --- | --- | --- | --- | --- | --- | --- | --- | --- | --- | --- |
|  | β | 95% CI | *P* | β | 95% CI | *P* | β | 95% CI | *P* | β | 95% CI | *P* |
| Residential distance | 1679.2 | 748.6 to 2609.8 | 0.001 | 1731.0 | 681.7 to 2780.2 | 0.001 | 1000.1 | -135.8 to 2135.9 | 0.08 | 2731.0 | 967.2 to 4494.7 | 0.002 |
| Nitrogen dioxide air pollution | -2383.0 | -3464.0 to -1301.0 | 0.001 | -2498.0 | -3717.0 to -1278.0 | 0.001 | -1791.0 | -3112.0 to -470.7 | 0.008 | -4289.0 | -6339.0 to -2240.0 | 0.001 |
| Nitrogen oxides air pollution | -2027.0 | -3102.0 to -952.7 | 0.001 | -1997.0 | -3209.0 to -785.3 | 0.001 | -1303.0 | -2615.0 to 8.7 | 0.05 | -3300.0 | -5338.0 to -1263.0 | 0.001 |
| PM_10_ air pollution | -1676.0 | -2650.0 to -702.4 | 0.001 | -1898.0 | -2995.0 to -799.8 | 0.001 | -651.4 | -1840.0 to 537.4 | 0.29 | -2549.0 | -4395.0 to -703.0 | 0.007 |
| PM_2.5_ air pollution | -2071.0 | -3068.0 to -1075.0 | 0.001 | -2013.0 | -3136.0 to -889.0 | 0.001 | -1313.0 | -2529.0 to -96.6 | 0.03 | -3325.0 | -5214.0 to -1437.0 | 0.001 |

**^*^** Traffic exposures were analyzed on the scale of per SD increment. Multivariate linear regression was used to model relationships between traffic exposures and brain MRI measures. Adjusted covariates included age, sex, ethnic background, education, current smoking, alcohol intake, physical activity, obesity, depressed mood, hypertension, diabetes, stroke, coronary heart disease, as well as brain MRI measuring positions. Socioeconomic status variables including the household income and the multiple deprivation index at the at the small area level were also adjusted.

**Appendix Table 14. Associations between residential distance to the nearest major road and brain structure measures, based on the IPW sample.**

| **Traffic exposures, per SD ^*^** | **Peripheral cortical grey matter volume (mm^3^)** | | | **Grey matter**  **volume (mm^3^)** | | | **White matter**  **volume (mm^3^)** | | | **Total brain**  **volume (mm^3^)** | | |
| --- | --- | --- | --- | --- | --- | --- | --- | --- | --- | --- | --- | --- |
|  | β | 95% CI | *P* | β | 95% CI | *P* | β | 95% CI | *P* | β | 95% CI | *P* |
| Residential distance | 1478.2 | 687.2 to 2269.1 | 0.001 | 1710.8 | 819.1 to 2602.5 | 0.001 | 875.0 | -100.7 to 1850.7 | 0.08 | 2585.7 | 1104.8 to 4066.7 | 0.001 |
| Nitrogen dioxide air pollution | -2008.0 | -3020.0 to -995.1 | 0.001 | -2646.0 | -3787.0 to -1505.0 | 0.001 | -1310.0 | -2559.0 to -60.8 | 0.04 | -3956.0 | -5852.0 to -2061.0 | 0.001 |
| Nitrogen oxides air pollution | -1695.0 | -2714.0 to -675.6 | 0.001 | -2022.0 | -3171.0 to -873.1 | 0.001 | -731.7 | -1989.0 to 525.5 | 0.25 | -2754.0 | -4662.0 to -845.1 | 0.005 |
| PM_10_ air pollution | -1270.0 | -2175.0 to -365.4 | 0.006 | -1856.0 | -2876.0 to -836.4 | 0.001 | 245.4 | -870.5 to 1361.3 | 0.67 | -1611.0 | -3305.0 to 84.3 | 0.06 |
| PM_2.5_ air pollution | -1731.0 | -2708.0 to -755.2 | 0.001 | -2092.0 | -3193.0 to -991.8 | 0.001 | -618.8 | -1823.0 to 585.6 | 0.31 | -2711.0 | -4539.0 to -882.8 | 0.004 |

**^*^** Traffic exposures were analyzed on the scale of per SD increment. Multivariate linear regression was used to model relationships between traffic exposures and brain MRI measures. Adjusted covariates included age, sex, ethnic background, education, current smoking, alcohol intake, physical activity, obesity, depressed mood, hypertension, diabetes, stroke, coronary heart disease, as well as brain MRI measuring positions. Analysis was conducted based on the IPW sample to address the potential selection bias.

**Appendix Table 15. Association between residential distance to the nearest major road, traffic-related air pollution with dementia incidence, further controlling for *APOE* ε4 carrier status.**

| **Traffic exposures, per SD ^*^** | **Dementia incidence (case/n=7000/460 901)** | | |
| --- | --- | --- | --- |
|  | HR | 95% CI | *P* |
| Residential distance to the nearest major road | 0.95 | 0.92-0.97 | 0.001 |
| Nitrogen dioxide air pollution | 1.09 | 1.06-1.11 | 0.001 |
| Nitrogen oxides air pollution | 1.07 | 1.04-1.09 | 0.001 |
| PM_10_ air pollution | 1.04 | 1.01-1.06 | 0.003 |
| PM_2.5_ air pollution | 1.08 | 1.06-1.11 | 0.001 |

**^*^** Traffic exposures were analyzed on the scale of per SD increment. Cox proportional hazard regression was used to model relationships between traffic exposures and dementia incidence. Age was used as the time scale variable. Adjusted covariates included sex, ethnic background, education, current smoking, alcohol intake, physical activity, obesity, depressed mood, hypertension, diabetes, stroke, and coronary heart disease. *APOE* ε4 carrier status was also adjusted.

**Appendix Table 16. Association between residential distance to the nearest major road, traffic-related air pollution with dementia incidence, further controlling for dementia polygenic risk category.**

| **Traffic exposures, per SD ^*^** | **Dementia incidence (case/n=7000/460 901)** | | |
| --- | --- | --- | --- |
|  | HR | 95% CI | *P* |
| Residential distance to the nearest major road | 0.95 | 0.92-0.98 | 0.001 |
| Nitrogen dioxide air pollution | 1.09 | 1.06-1.11 | 0.001 |
| Nitrogen oxides air pollution | 1.07 | 1.04-1.09 | 0.001 |
| PM_10_ air pollution | 1.04 | 1.01-1.06 | 0.003 |
| PM_2.5_ air pollution | 1.08 | 1.06-1.11 | 0.001 |

**^*^** Traffic exposures were analyzed on the scale of per SD increment. Cox proportional hazard regression was used to model relationships between traffic exposures and dementia incidence. Age was used as the time scale variable. Adjusted covariates included sex, ethnic background, education, current smoking, alcohol intake, physical activity, obesity, depressed mood, hypertension, diabetes, stroke, and coronary heart disease. Dementia polygenic risk category was also adjusted.

**Appendix Table 17. Association between residential distance to the nearest major road, traffic-related air pollution with dementia incidence, further stratified by *APOE* ε4 carrier status.**

| **Traffic exposures, per SD ^*^** | **Non-*APOE* ε4 carrier (case/n=4040/352 983)** | |  | ***APOE* ε4 carrier (case/n=2960/107 918)** | | ***P* for interaction** |
| --- | --- | --- | --- | --- | --- | --- |
|  | HR | 95% CI |  | HR | 95% CI |  |
| Residential distance to the nearest major road | 0.93 | 0.89-0.97 |  | 0.96 | 0.92-1.01 | 0.21 |
| Nitrogen dioxide air pollution | 1.10 | 1.07-1.13 |  | 1.08 | 1.04-1.12 | 0.58 |
| Nitrogen oxides air pollution | 1.08 | 1.05-1.11 |  | 1.05 | 1.01-1.09 | 0.25 |
| PM_10_ air pollution | 1.06 | 1.03-1.10 |  | 1.01 | 0.97-1.04 | 0.03 |
| PM_2.5_ air pollution | 1.11 | 1.07-1.14 |  | 1.06 | 1.02-1.10 | 0.05 |

**^*^** Traffic exposures were analyzed on the scale of per SD increment. Cox proportional hazard regression was used to model relationships between traffic exposures and dementia incidence. Age was used as the time scale variable. Adjusted covariates included sex, ethnic background, education, current smoking, alcohol intake, physical activity, obesity, depressed mood, hypertension, diabetes, stroke, and coronary heart disease.

**Appendix Table 18. Association between residential distance to the nearest major road, traffic-related air pollution with dementia incidence, further stratified by dementia polygenic risk category.**

| **Traffic exposures, per SD ^a^** | **Low-to-intermediate risk (case/n=4249/371 721)** | |  | **High risk (case/n=2751/89180)** | | ***P* for interaction** |
| --- | --- | --- | --- | --- | --- | --- |
|  | HR | 95% CI |  | HR | 95% CI |  |
| Residential distance to the nearest major road | 0.94 | 0.90-0.97 |  | 0.96 | 0.92-1.10 | 0.48 |
| Nitrogen dioxide air pollution | 1.10 | 1.07-1.13 |  | 1.08 | 1.04-1.12 | 0.49 |
| Nitrogen oxides air pollution | 1.07 | 1.04-1.10 |  | 1.07 | 1.03-1.11 | 0.89 |
| PM_10_ air pollution | 1.06 | 1.03-1.09 |  | 1.01 | 0.97-1.05 | 0.08 |
| PM_2.5_ air pollution | 1.10 | 1.07-1.14 |  | 1.06 | 1.02-1.10 | 0.08 |

**^*^** Traffic exposures were analyzed on the scale of per SD increment. Cox proportional hazard regression was used to model relationships between traffic exposures and dementia incidence. Age was used as the time scale variable. Adjusted covariates included sex, ethnic background, education, current smoking, alcohol intake, physical activity, obesity, depressed mood, hypertension, diabetes, stroke, and coronary heart disease.

**Appendix Table 19. Association between residential distance to the nearest major road, traffic-related air pollution with dementia incidence, further restricting to the pre-pandemic follow-up period.**

| **Traffic exposures, per SD ^*^** | **Dementia incidence (case/n=4887/460 901)** | | |
| --- | --- | --- | --- |
|  | HR | 95% CI | *P* |
| Residential distance to the nearest major road | 0.93 | 0.90-0.96 | 0.001 |
| Nitrogen dioxide air pollution | 1.09 | 1.06-1.12 | 0.001 |
| Nitrogen oxides air pollution | 1.07 | 1.04-1.10 | 0.001 |
| PM_10_ air pollution | 1.03 | 1.00-1.06 | 0.022 |
| PM_2.5_ air pollution | 1.09 | 1.06-1.12 | 0.001 |

**^*^** Traffic exposures were analyzed on the scale of per SD increment. Cox proportional hazard regression was used to model relationships between traffic exposures and dementia incidence. Age was used as the time scale variable. Adjusted covariates included sex, ethnic background, education, current smoking, alcohol intake, physical activity, obesity, depressed mood, hypertension, diabetes, stroke, and coronary heart disease.

**Appendix Table 20. Causal mediation analysis of the associations between residential distance to major roads with all-cause dementia incidence, estimated using the regression-based approach.**

| **Hypothesized mediators** | **Effect decomposition (95% CI) ^*^** | | | **Proportion mediated (%)** |
| --- | --- | --- | --- | --- |
|  | **Total effect** | **Natural direct effect** | **Natural indirect effect** |  |
| Nitrogen dioxide air pollution | 0.94 (0.92-0.97) | 0.97 (0.94-1.01) | 0.96 (0.95-0.97) | 61.2 (21.5, 99.9) |
| Nitrogen oxides air pollution | 0.94 (0.92-0.97) | 0.96 (0.93-0.99) | 0.97 (0.96-0.98) | 39.3 (11.0-67.7) |
| PM_10_ air pollution | 0.94 (0.92-0.97) | 0.95 (0.92-0.98) | 0.99 (0.98-1.00) | 12.1 (0.0-24.7) |
| PM_2.5_ air pollution | 0.94 (0.92-0.97) | 0.97 (0.94-1.00) | 0.96 (0.95-0.97) | 58.0 (19.6-96.3) |

**^*^** Traffic exposures were analyzed on the scale of per SD increment. Effect estimates were expressed as hazard ratios (HR). Adjusted covariates included sex, ethnic background, education, current smoking, alcohol intake, physical activity, obesity, depressed mood, hypertension, diabetes, stroke, and coronary heart disease.


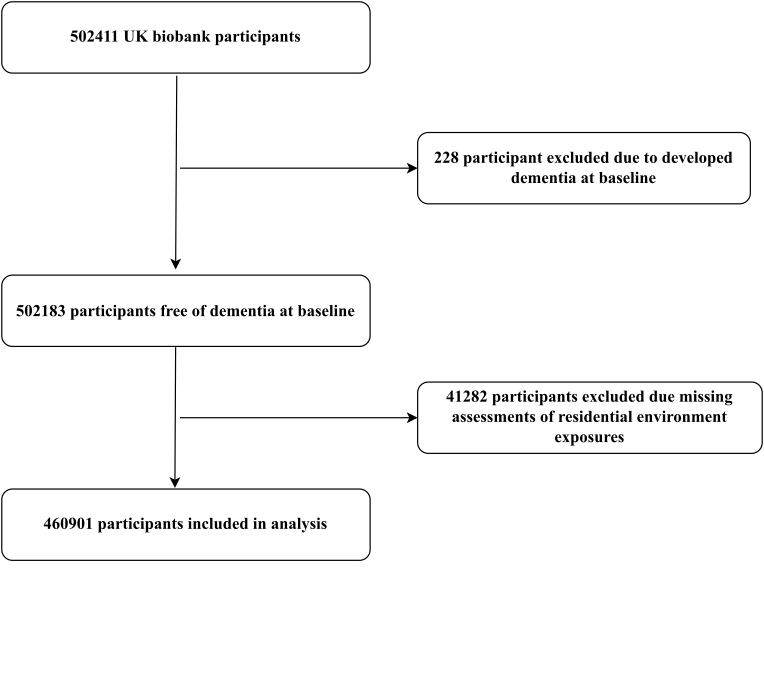


**Appendix Figure 1. Participants selection diagram.**


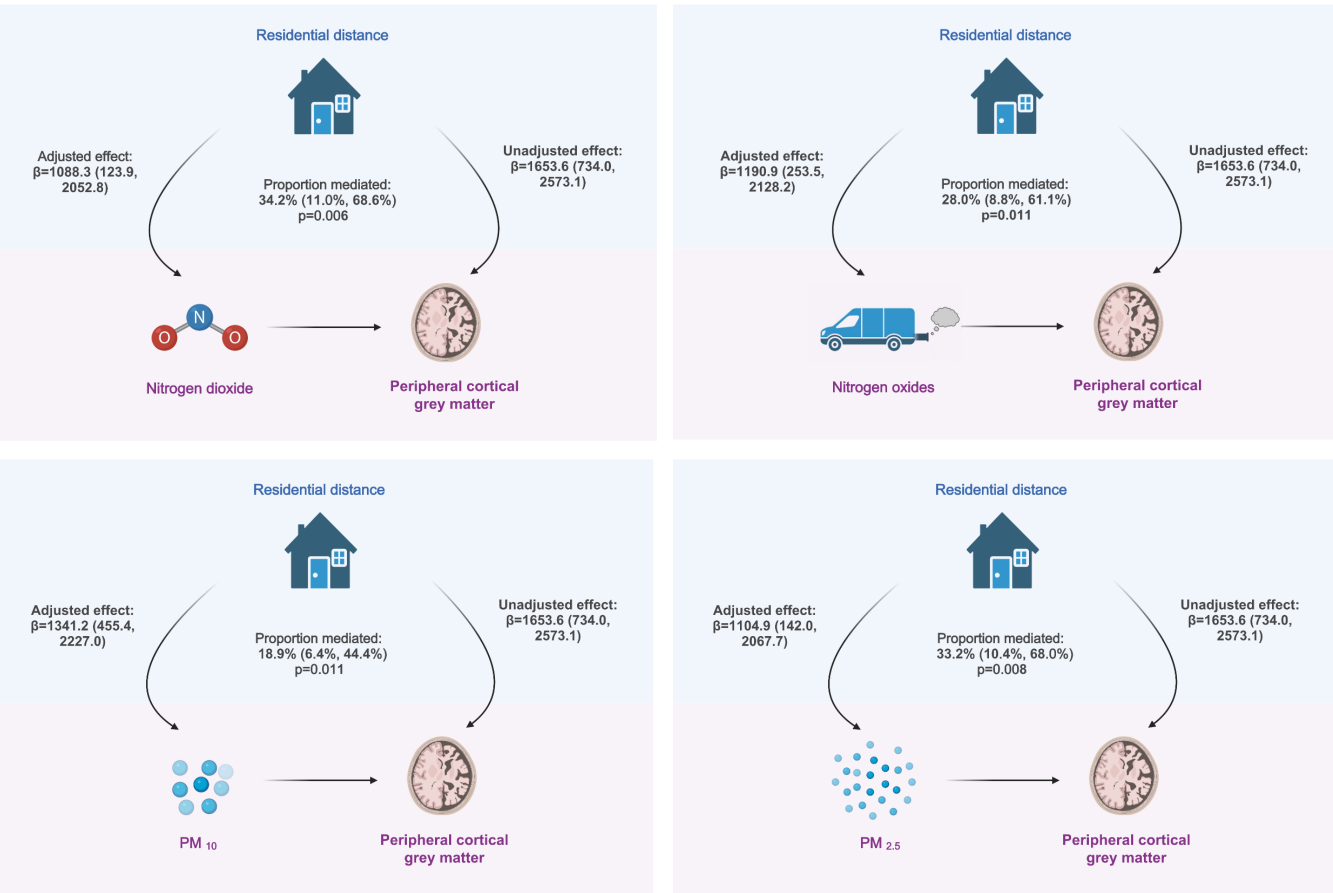


**Appendix Figure 2. Mediation analysis of the associations between residential distance to major roads with volume of peripheral cortical grey matter, by comparing the effect before and after adjusting for the hypothesized mediator of traffic-related air pollution.**

The unadjusted effect represents the estimate from the linear regression without adjusting for the hypothesized mediator, while the adjusted effect comes from the linear regression adjusting for the hypothesized mediator. Based on the difference between the unadjusted and adjusted effect, the proportion being mediated and hypothesis testing was calculated and conducted for each hypothesized mediator, respectively. Other adjusted covariates were identical to linear regression models in table 3. We did perform the mediation analysis for the white matter volume, owing to the lack of statistical significance regarding the association between residential distance to major roads with the outcome (**Table 3**).


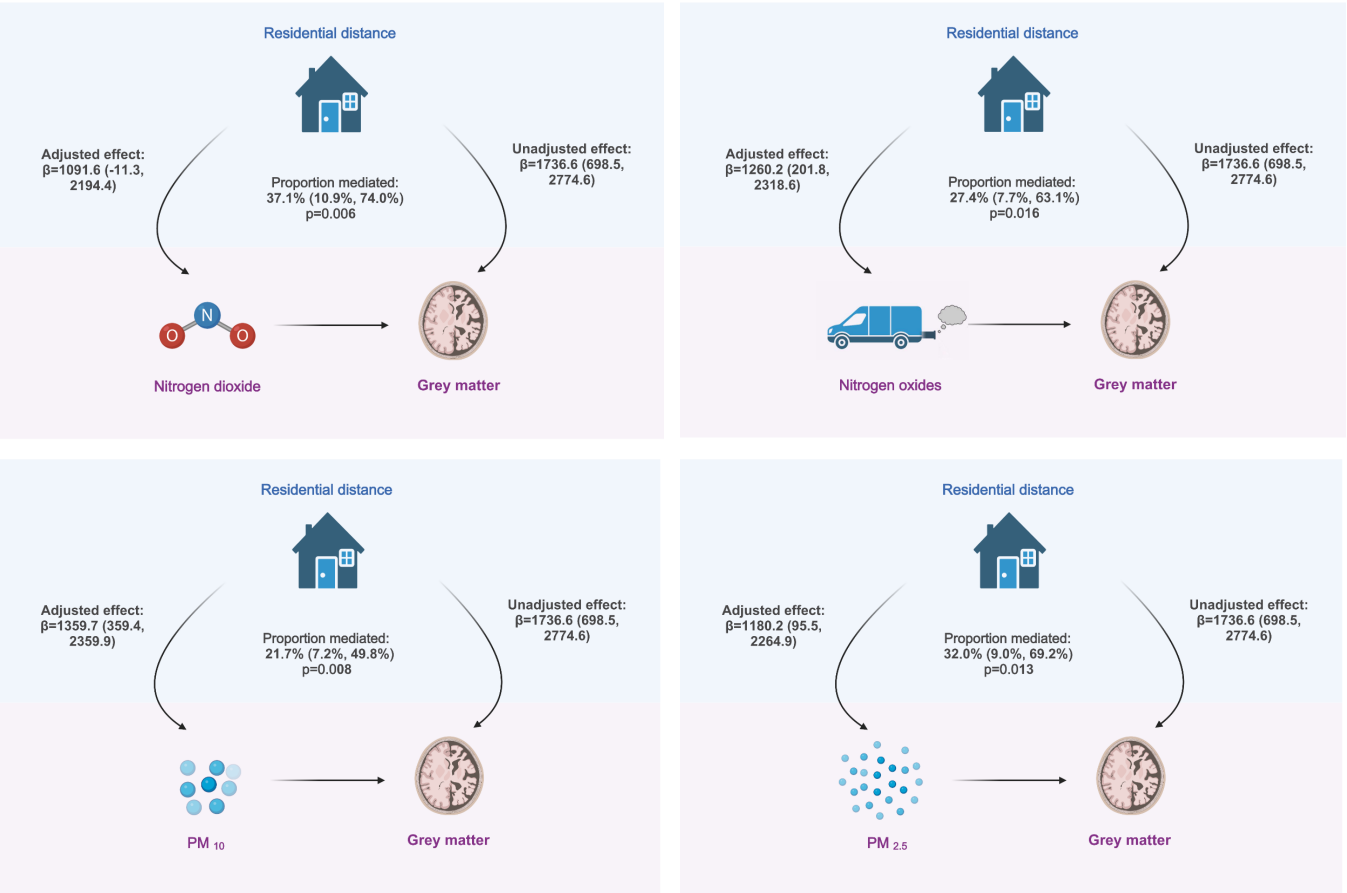


**Appendix Figure 3. Mediation analysis of the associations between residential distance to major roads with volume of grey matter, by comparing the effect before and after adjusting for the hypothesized mediator of traffic-related air pollution.**

The unadjusted effect represents the estimate from the linear regression without adjusting for the hypothesized mediator, while the adjusted effect comes from the linear regression adjusting for the hypothesized mediator. Based on the difference between the unadjusted and adjusted effect, the proportion being mediated and hypothesis testing was calculated and conducted for each hypothesized mediator, respectively. Other adjusted covariates were identical to linear regression models in table 3. We did perform the mediation analysis for the white matter volume, owing to the lack of statistical significance regarding the association between residential distance to major roads with the outcome (**Table 3**).


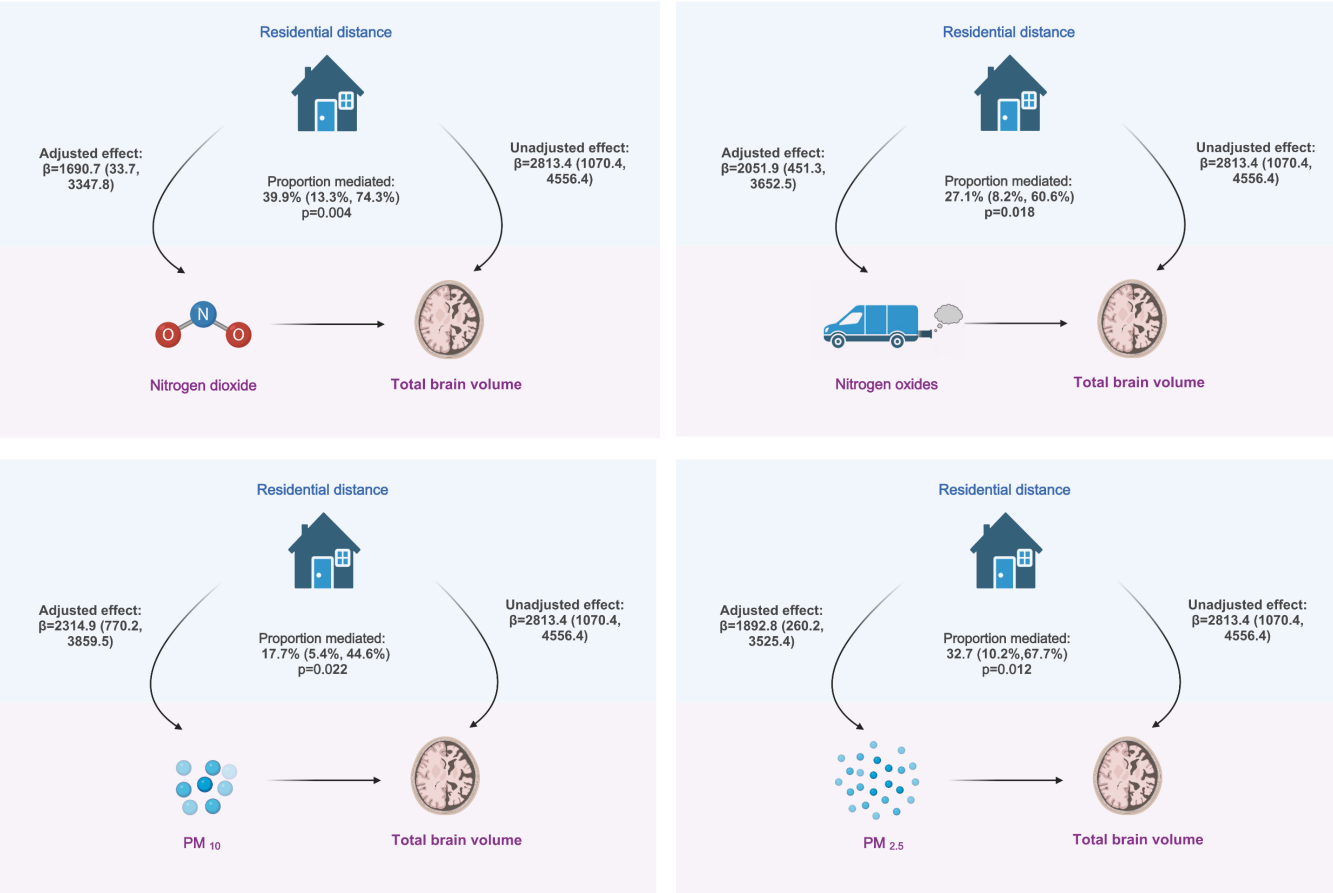


**Appendix Figure 4. Mediation analysis of the associations between residential distance to major roads with volume of total brain, by comparing the effect before and after adjusting for the hypothesized mediator of traffic-related air pollution.**

The unadjusted effect represents the estimate from the linear regression without adjusting for the hypothesized mediator, while the adjusted effect comes from the linear regression adjusting for the hypothesized mediator. Based on the difference between the unadjusted and adjusted effect, the proportion being mediated and hypothesis testing was calculated and conducted for each hypothesized mediator, respectively. Other adjusted covariates were identical to linear regression models in table 3. We did perform the mediation analysis for the white matter volume, owing to the lack of statistical significance regarding the association between residential distance to major roads with the outcome (**Table 3**).
